# Supplementary figures and images for: Hydroxyurea modulates thiol–disulfide homeostasis in the yeast endoplasmic reticulum
Source: Life Sci Alliance. 2025 Jun 20;8(8):e202503225. doi: 10.26508/lsa.202503225 (PMC12181674; doi:10.26508/lsa.202503225)

**Fig. 1A**

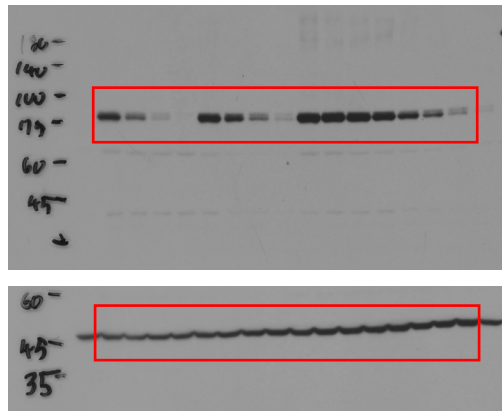

**Fig. 1B**

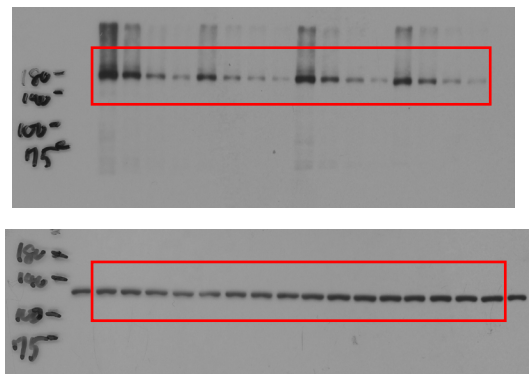

**Fig. 1C**

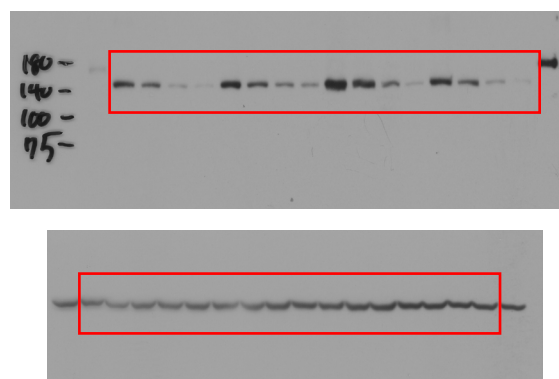

Supplement: Supplementary file 1 [file LSA-2025-03225_SdataF1.1.pdf]

Fig. S2A

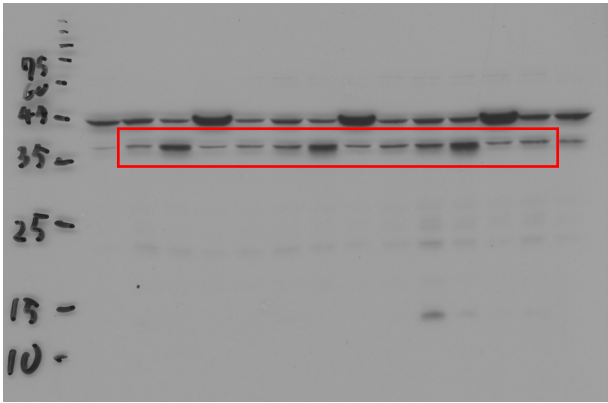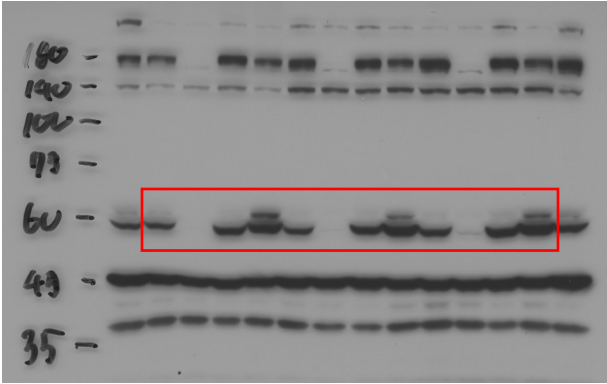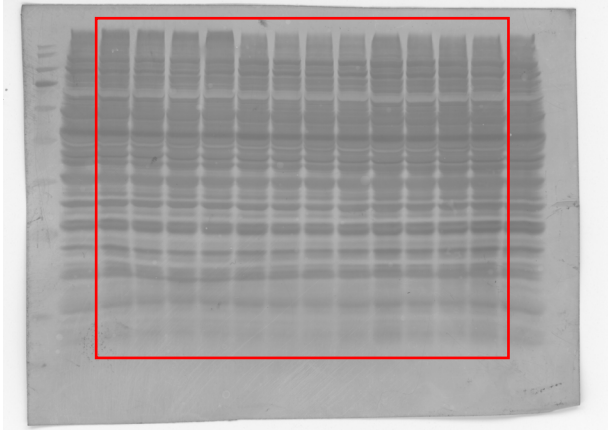

Fig. S2B

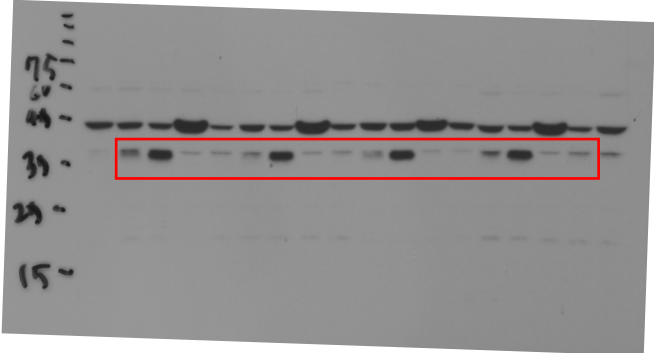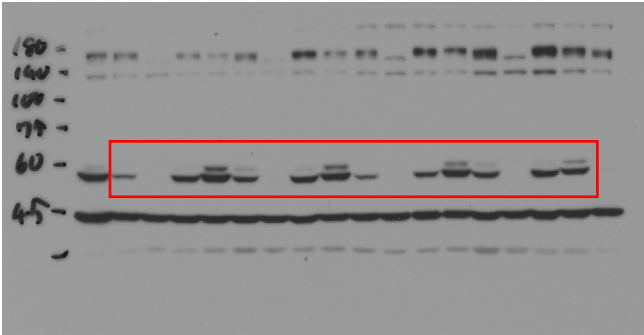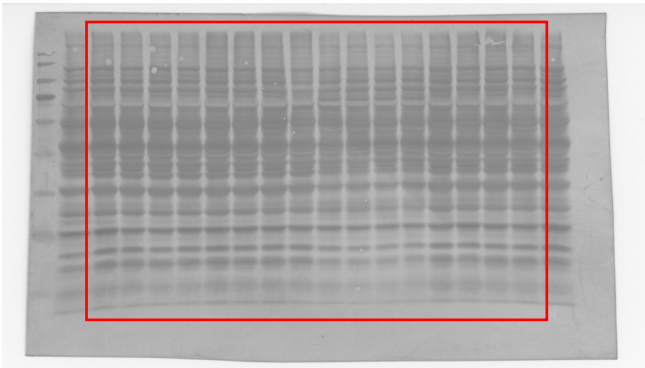

Supplement: Supplementary file 6 [file LSA-2025-03225_SdataFS2.pdf]

**Fig. S3**

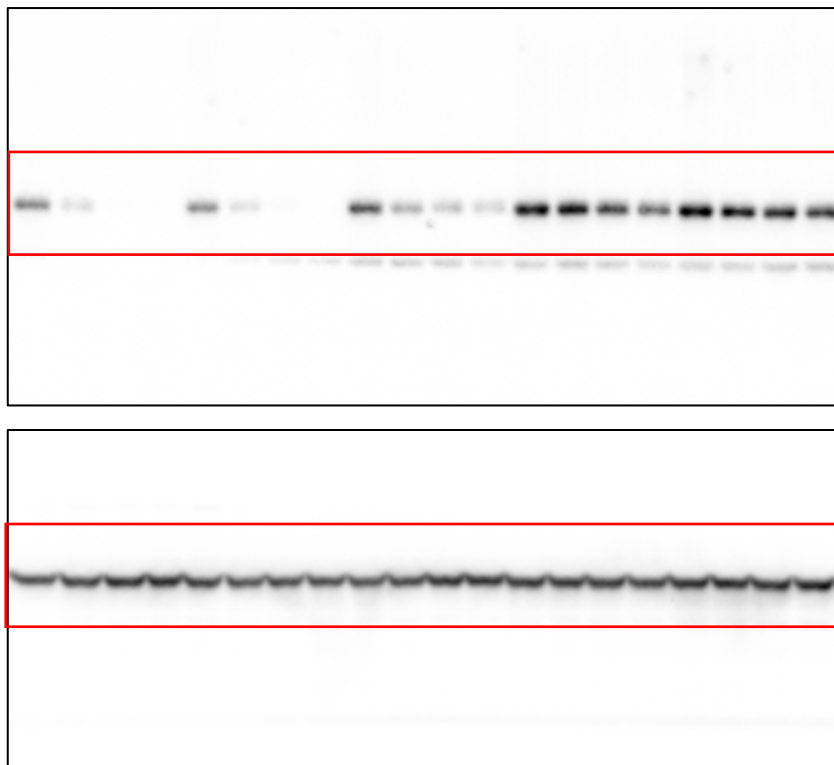

Supplement: Supplementary file 7 [file LSA-2025-03225_SdataFS3.1.pdf]

**Fig. S4A**

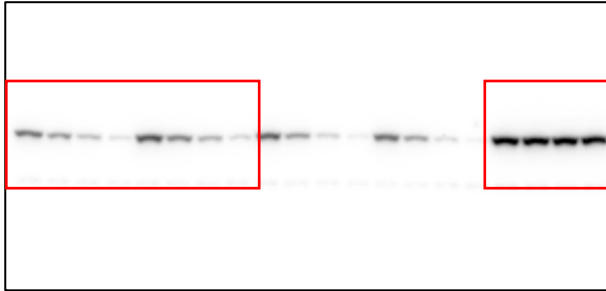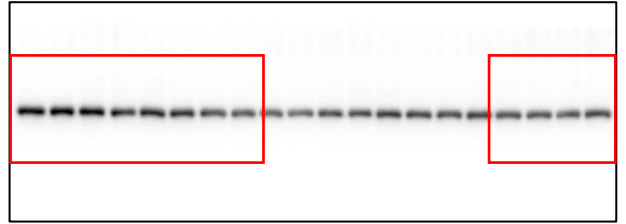

**Fig. S4B**

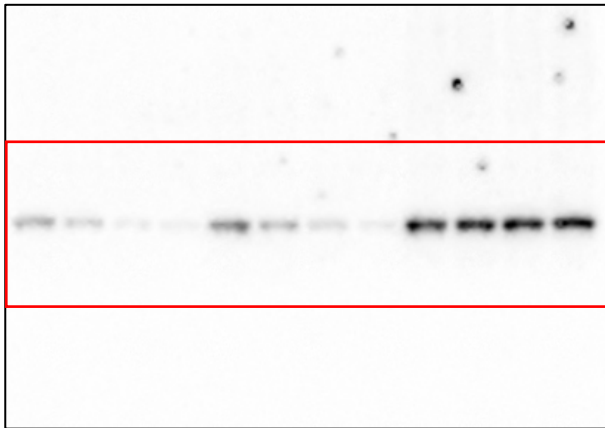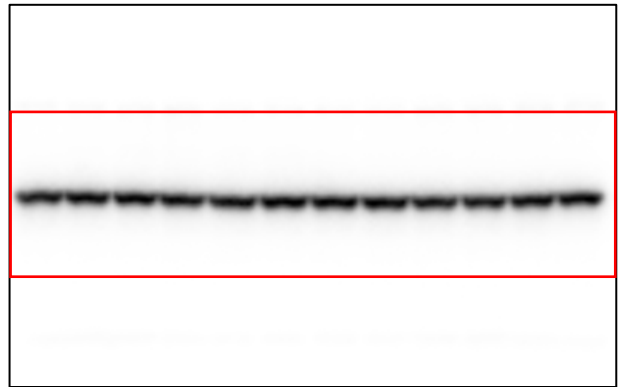

**Fig. S4C**

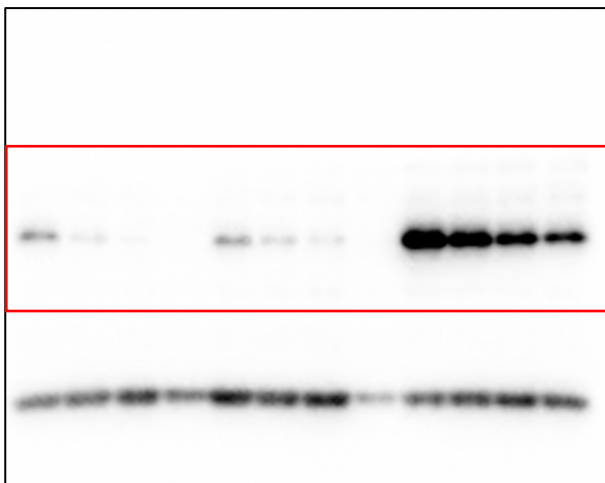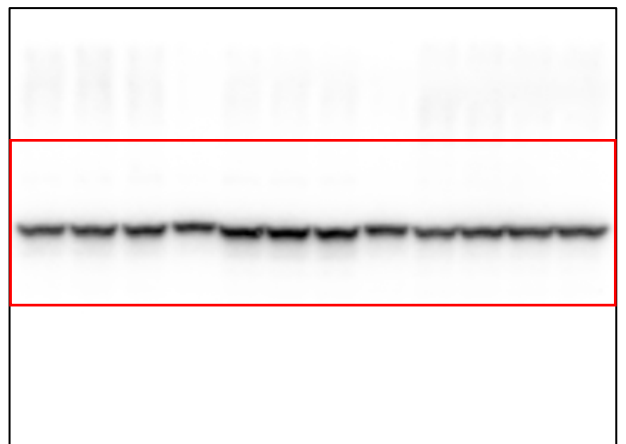

Supplement: Supplementary file 9 [file LSA-2025-03225_SdataFS4.1.pdf]

Fig. 2A

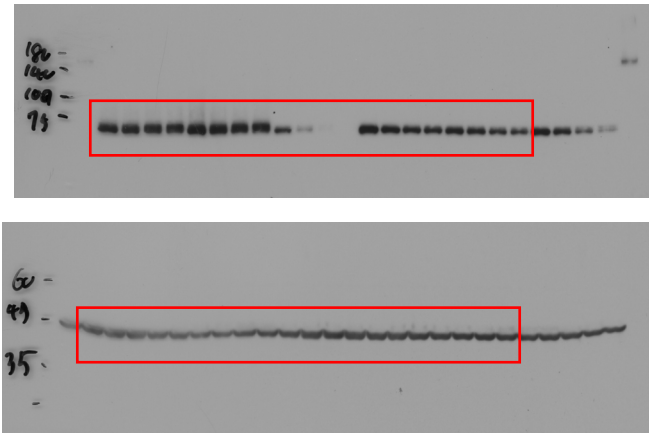

Fig. 2B

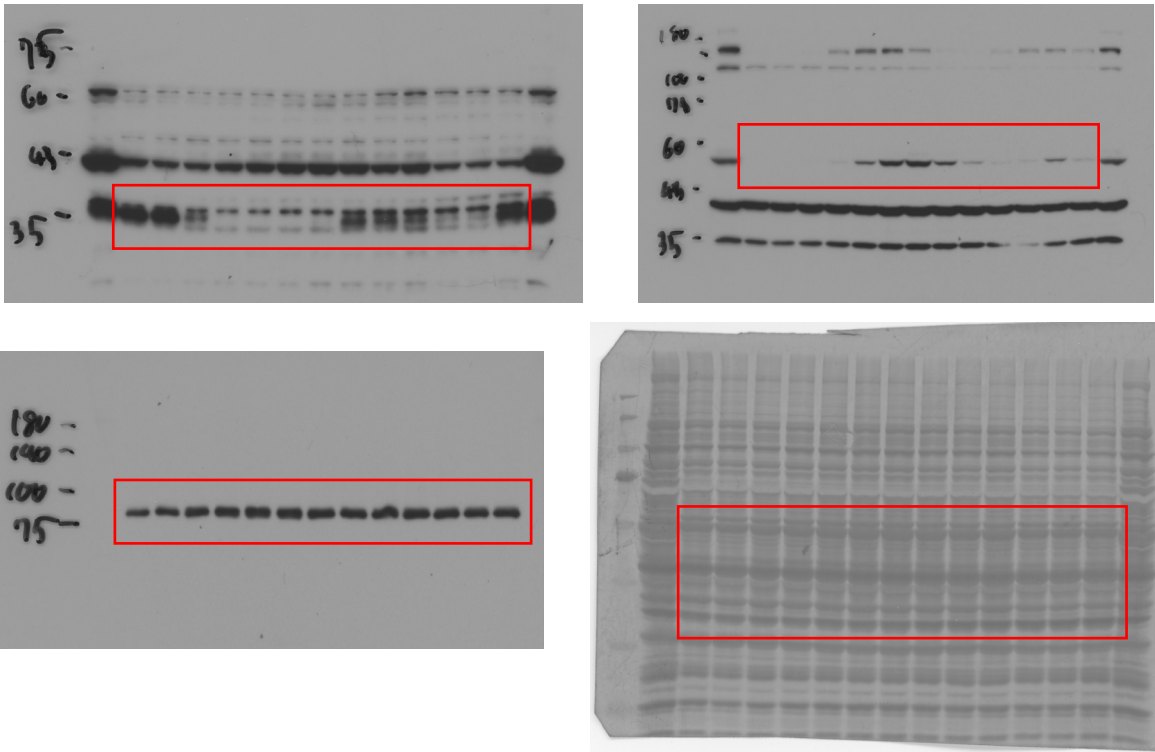

Fig. 2C

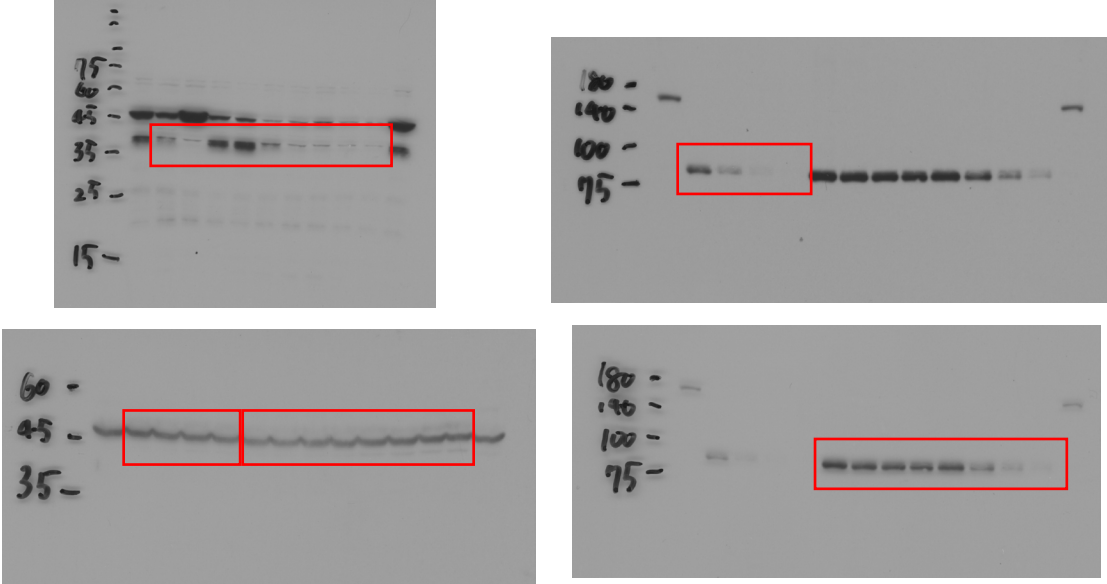

Fig. 2D

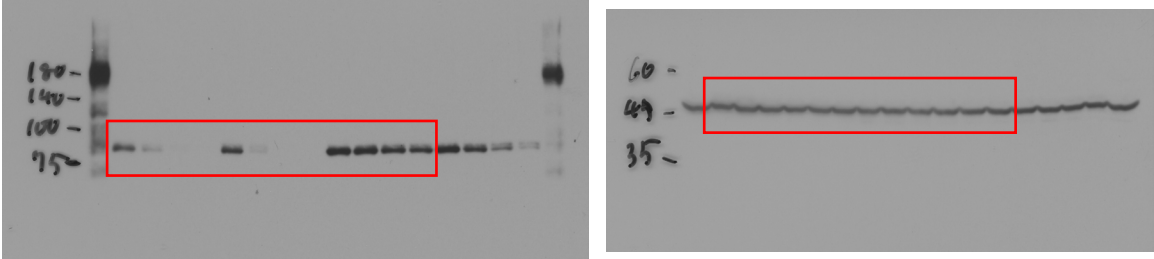

Fig. 2E

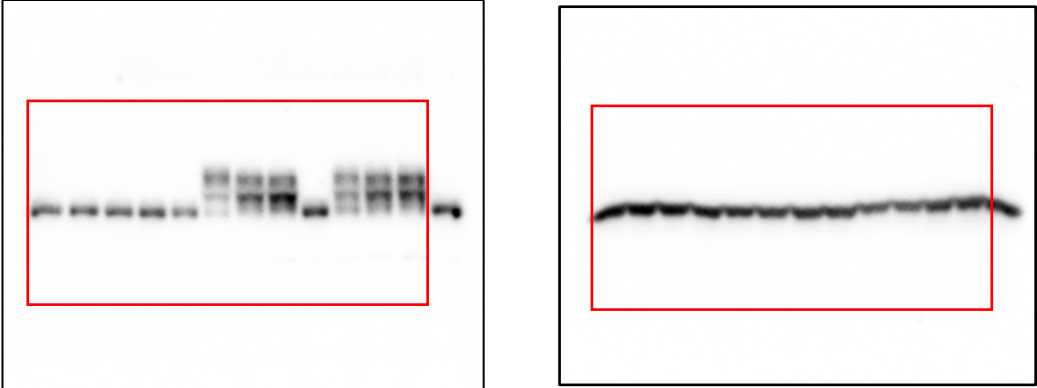

Supplement: Supplementary file 11 [file LSA-2025-03225_SdataF2.1.pdf]

Fig. S5B

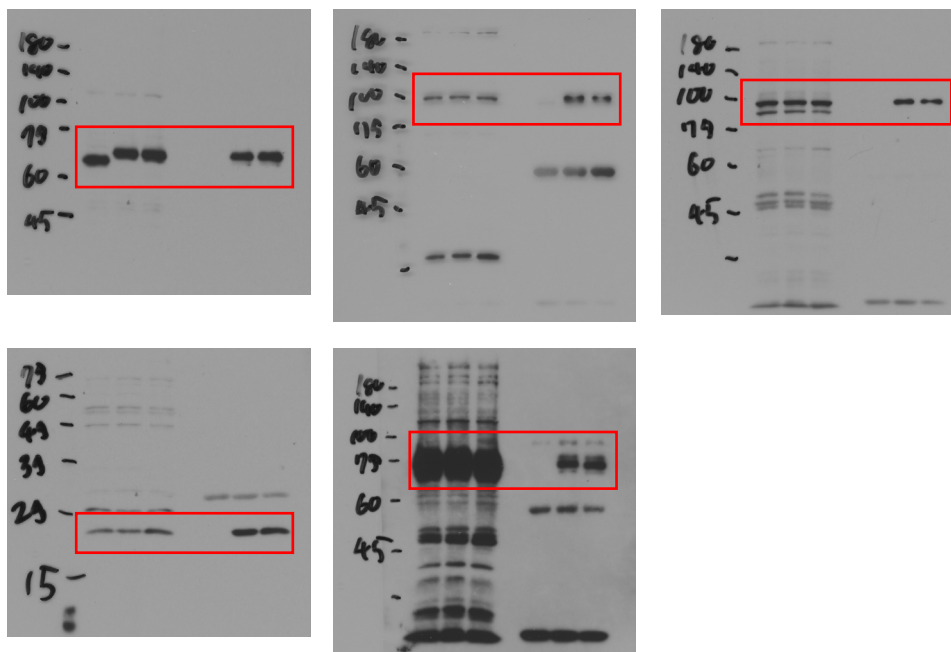

Fig. S5C

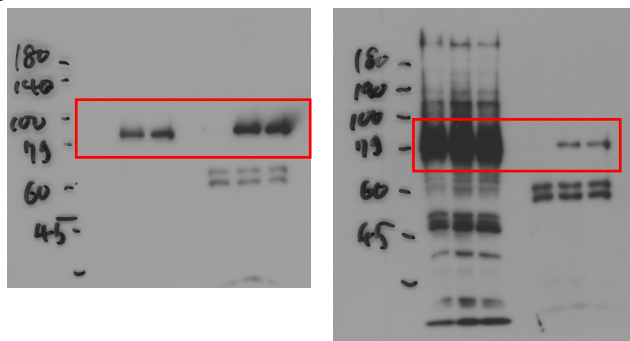

Fig. S5E

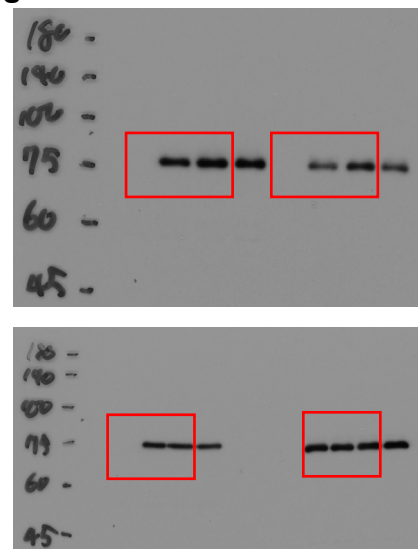

Fig. S5D

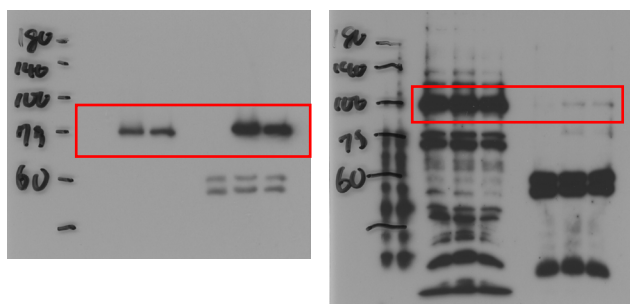

Supplement: Supplementary file 13 [file LSA-2025-03225_SdataFS5.pdf]

**Fig. S6A**

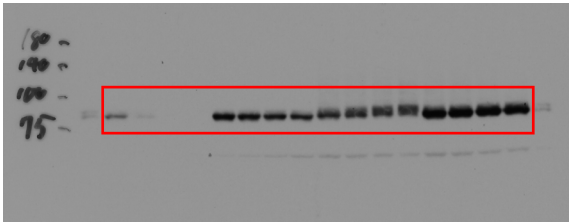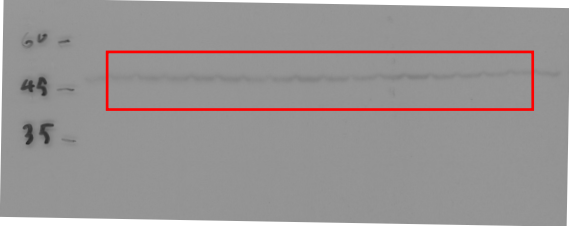

**Fig. S6B**

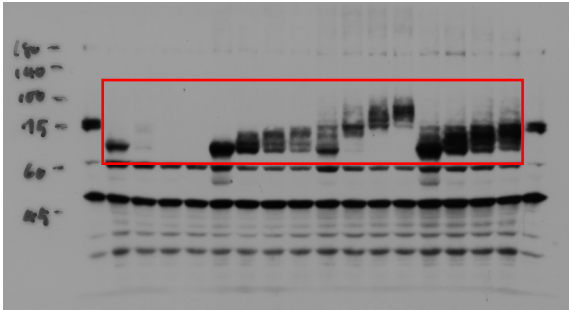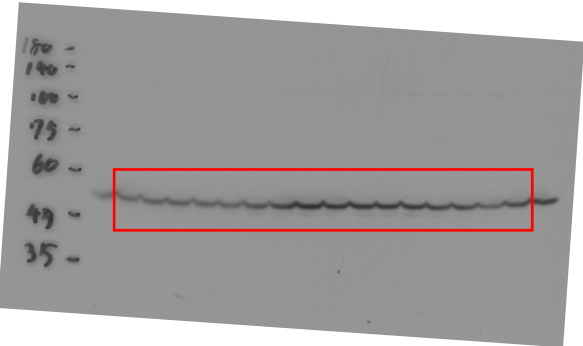

**Fig. S6C**

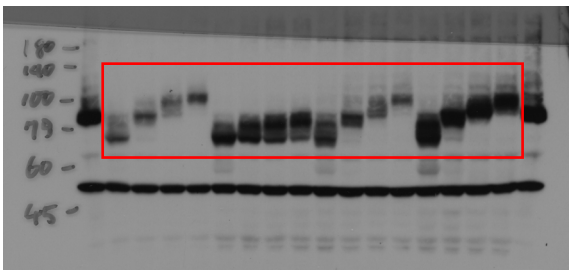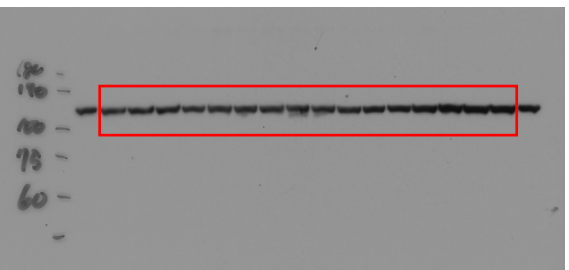

**Fig. S6D**

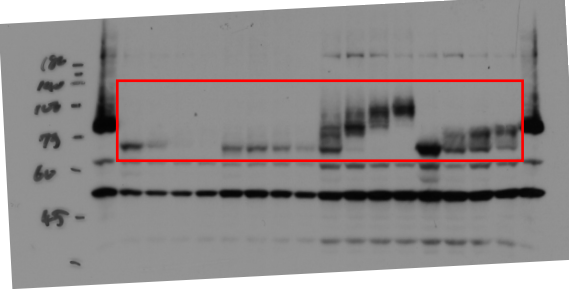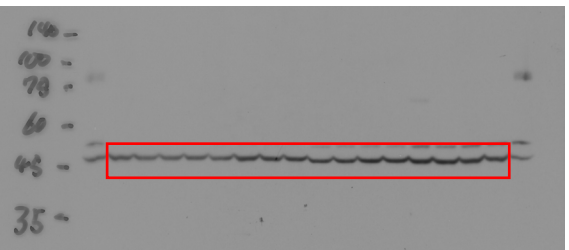

Supplement: Supplementary file 14 [file LSA-2025-03225_SdataFS6.pdf]

Fig. 3A

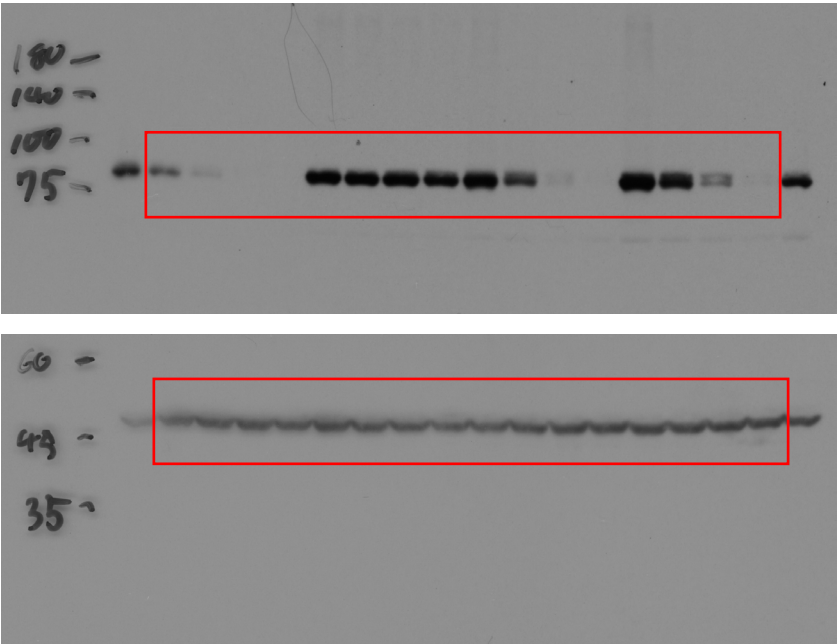

Fig. 3B

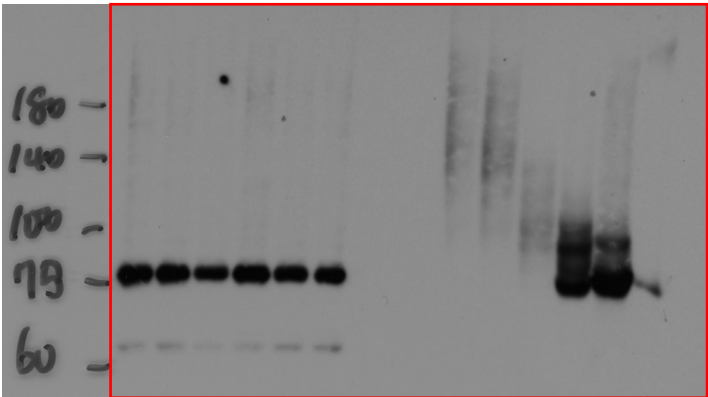

Fig. 3C

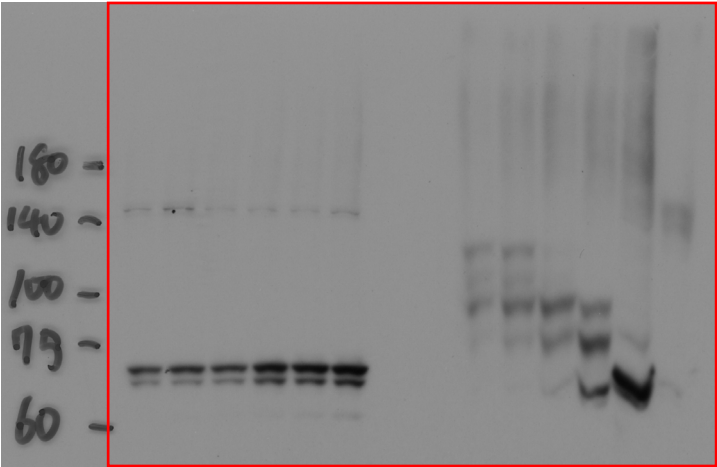

Supplement: Supplementary file 15 [file LSA-2025-03225_SdataF3.1.pdf]

**Fig. S7**

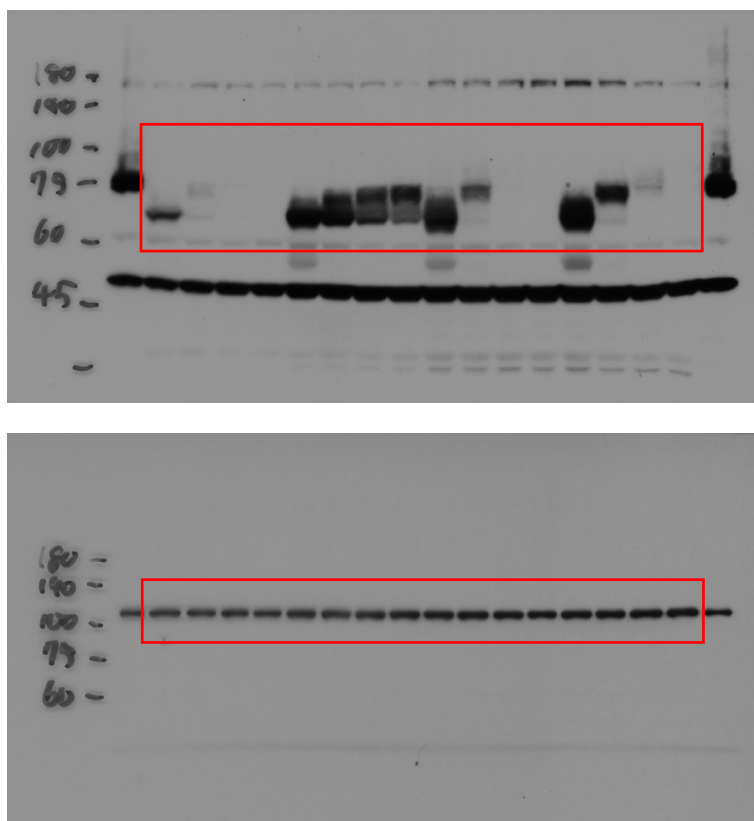

Supplement: Supplementary file 17 [file LSA-2025-03225_SdataFS7.1.pdf]

**Fig. S8A**

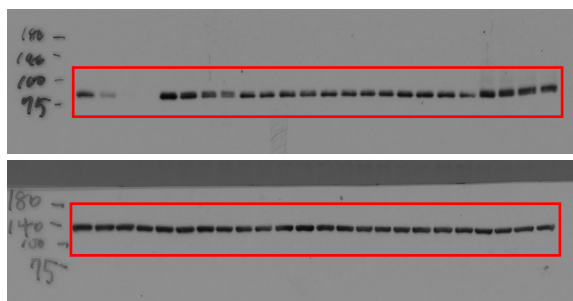

**Fig. S8B**

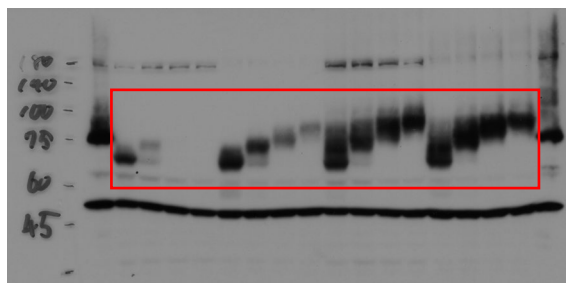

**Fig. S8C**

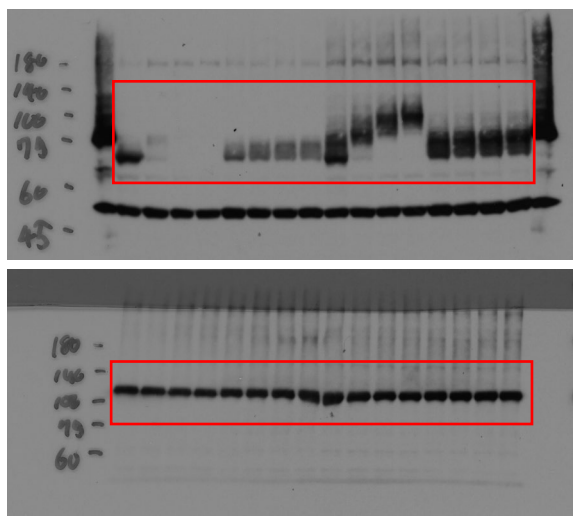

**Fig. S8D**

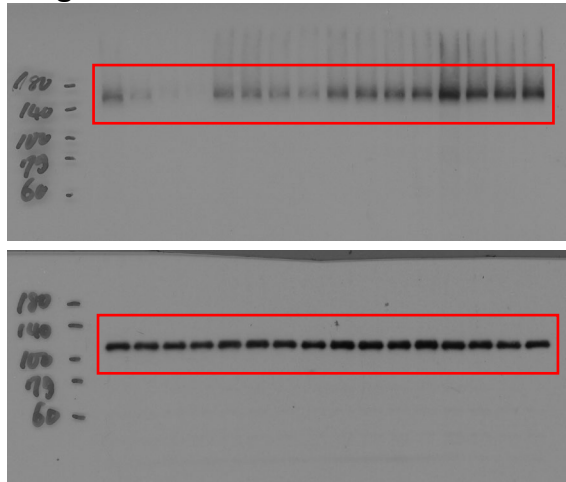

**Fig. S8E**

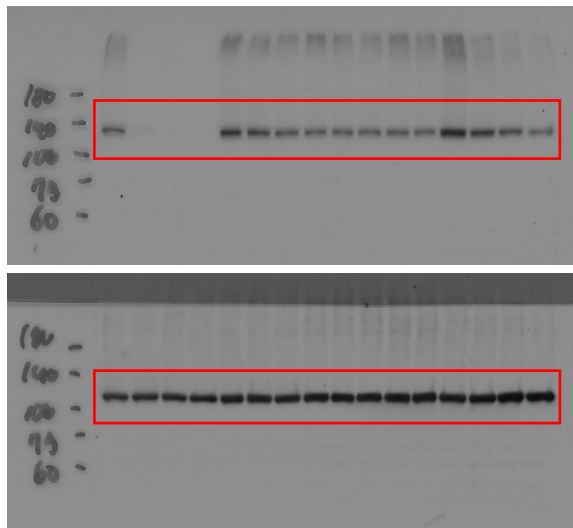

**Fig. S8F**

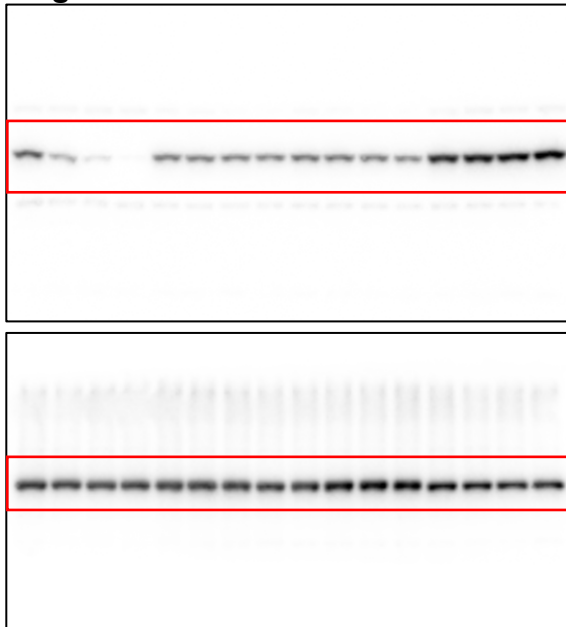

Supplement: Supplementary file 19 [file LSA-2025-03225_SdataFS8.1.pdf]

**Fig. S9A**

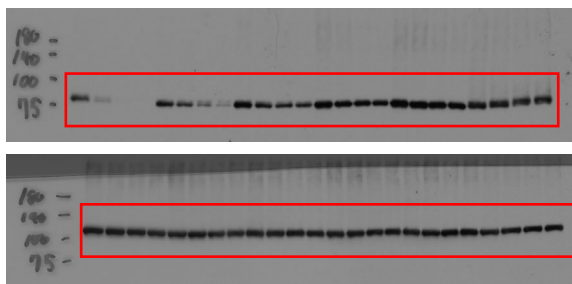

**Fig. S9B**

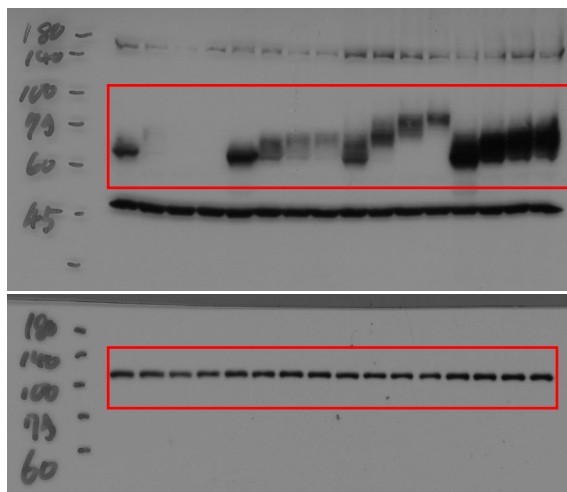

**Fig. S9C**

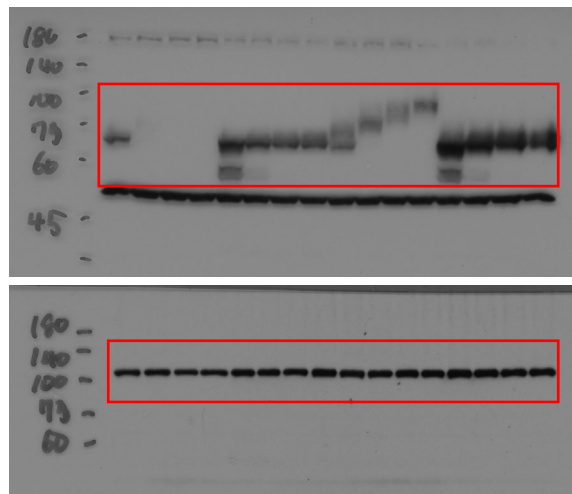

**Fig. S9D**

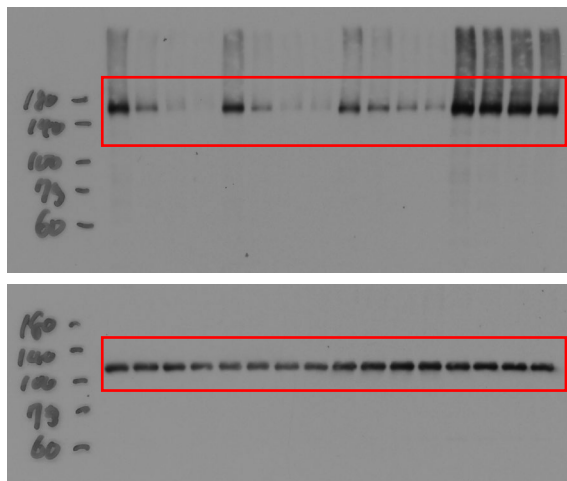

**Fig. S9E**

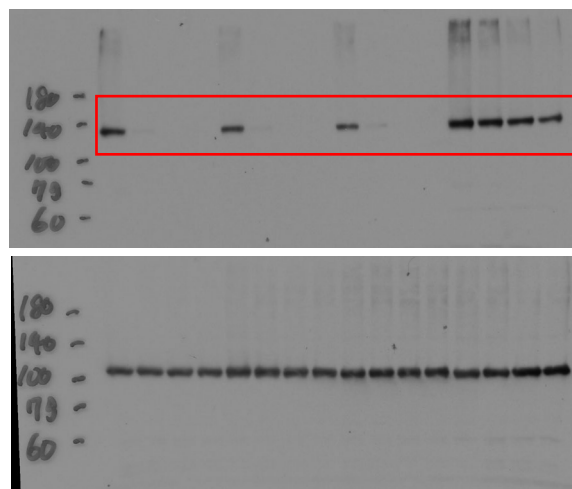

**Fig. S9F**

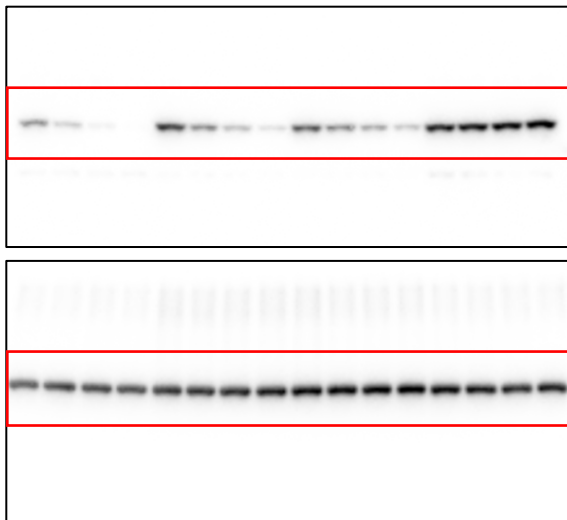

Supplement: Supplementary file 21 [file LSA-2025-03225_SdataFS9.1.pdf]

**Fig. S10B**

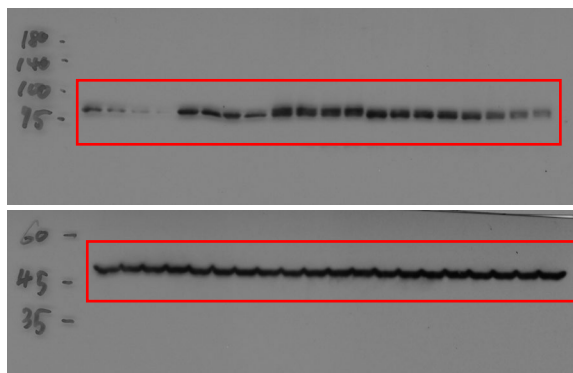

**Fig. S10C**

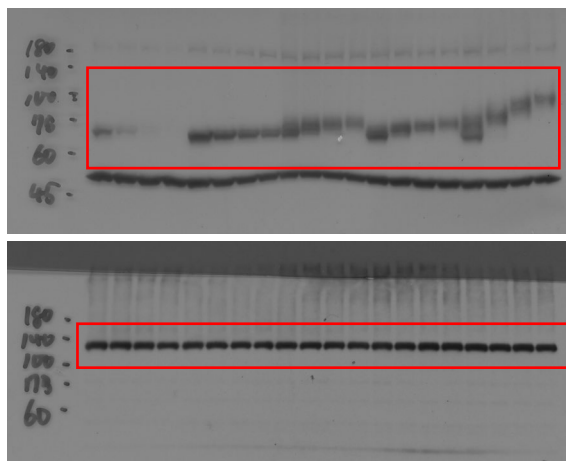

**Fig. S10D**

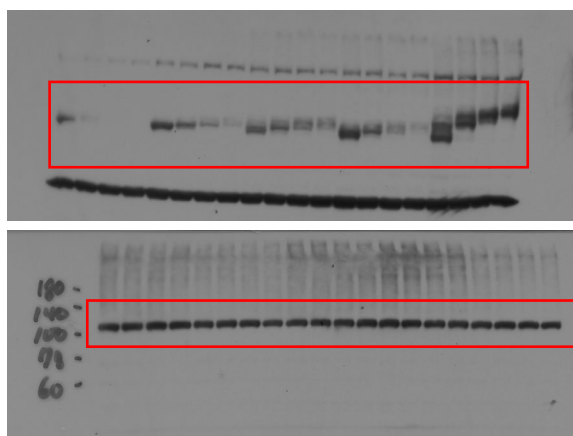

**Fig. S10E**

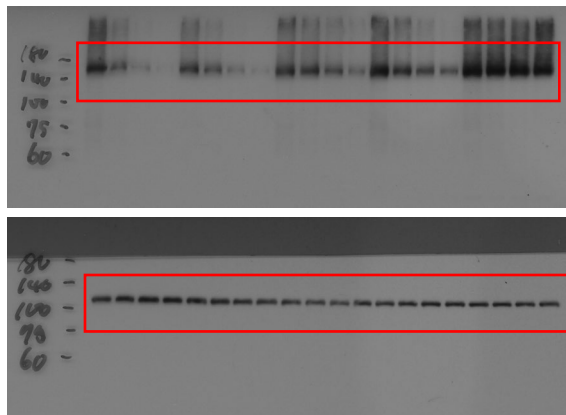

**Fig. S10F**

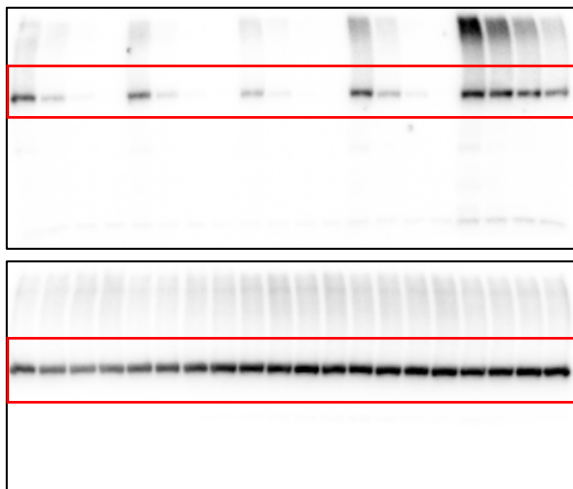

Supplement: Supplementary file 23 [file LSA-2025-03225_SdataFS10.1.pdf]

**Fig. S12A**

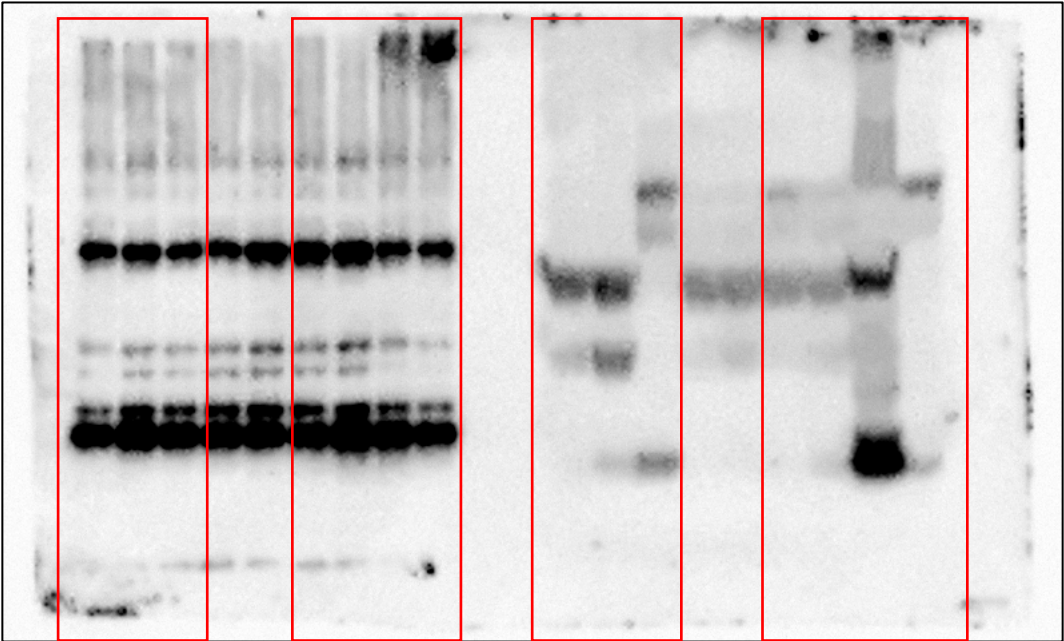

**Fig. S12B**

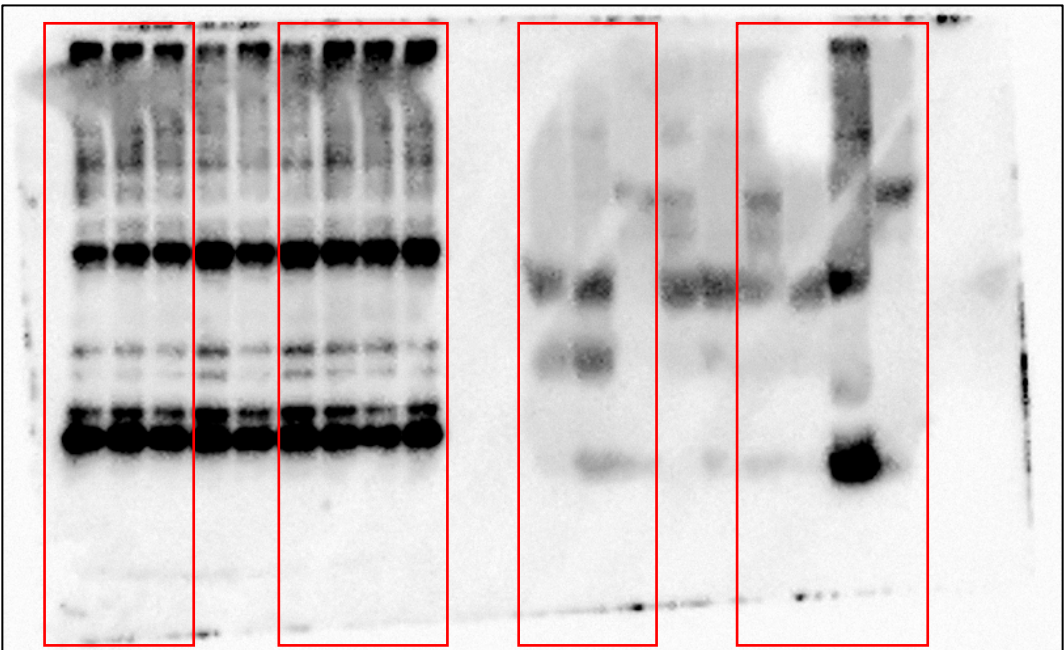

Supplement: Supplementary file 25 [file LSA-2025-03225_SdataFS12.pdf]

**Fig. S13A: HU**

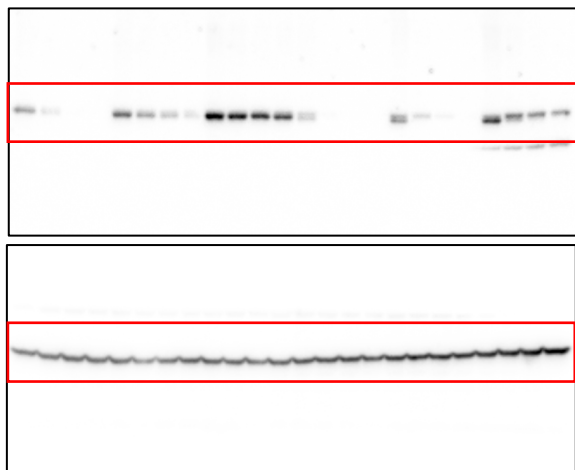

**Fig. S13A: diamide**

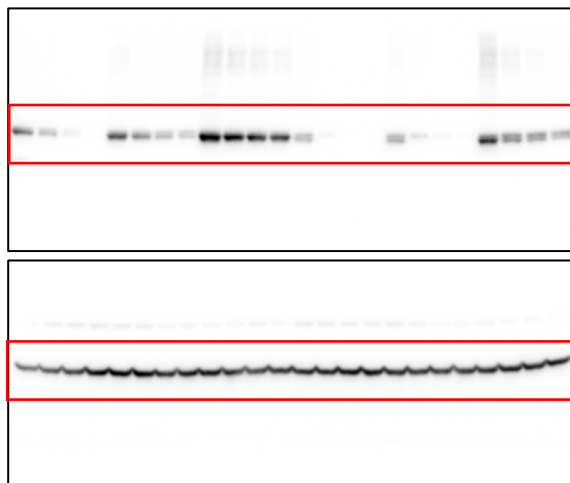

**Fig. S13A: H<sub>2</sub>O<sub>2</sub>**

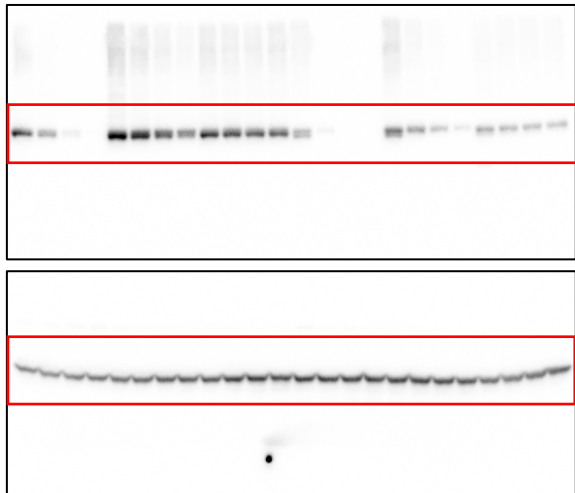

**Fig. S13B left**

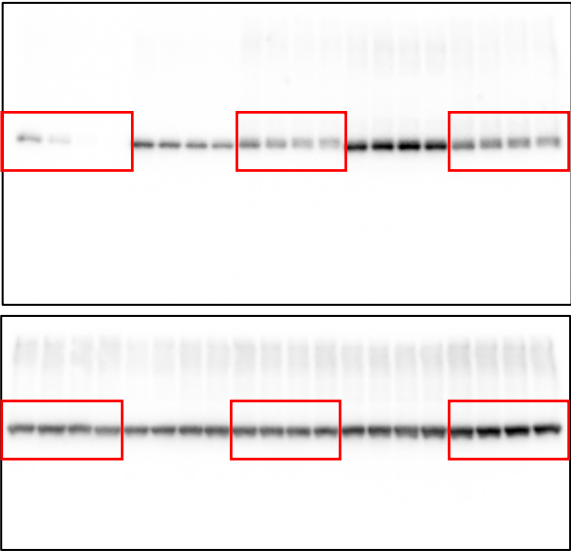

**Fig. S13B right**

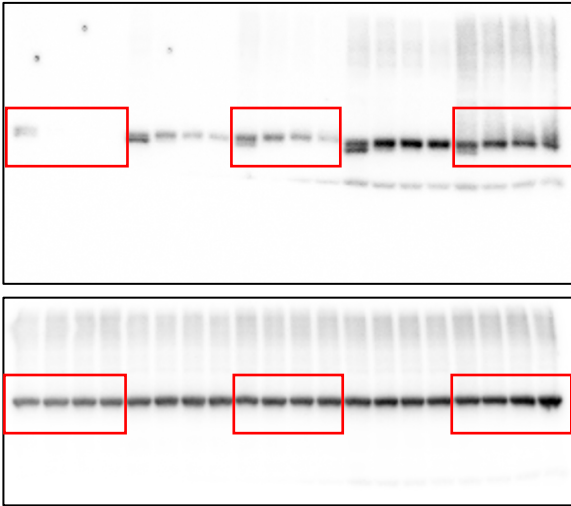

**Fig. S13C**

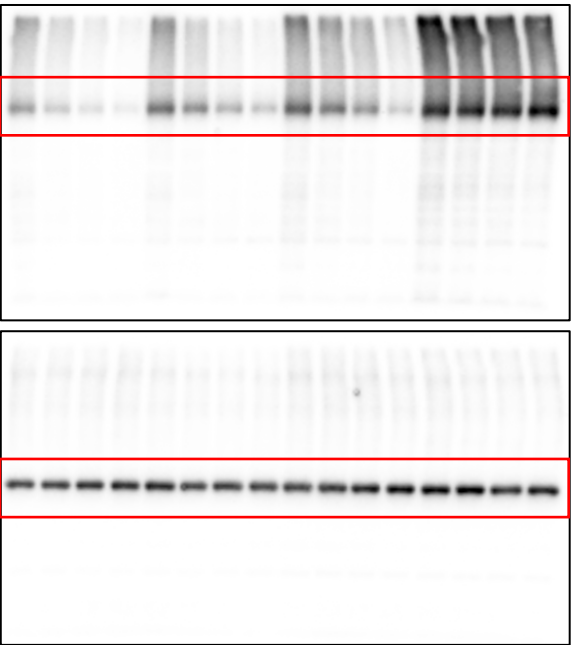

Supplement: Supplementary file 26 [file LSA-2025-03225_SdataFS13.1.pdf]

**Fig. 4A**

HU 0 mM 26°C

HU 0 mM 37°C

HU 10 mM 26°C

HU 10 mM 37°C

HU 50 mM 26°C

HU 50 mM 37°C

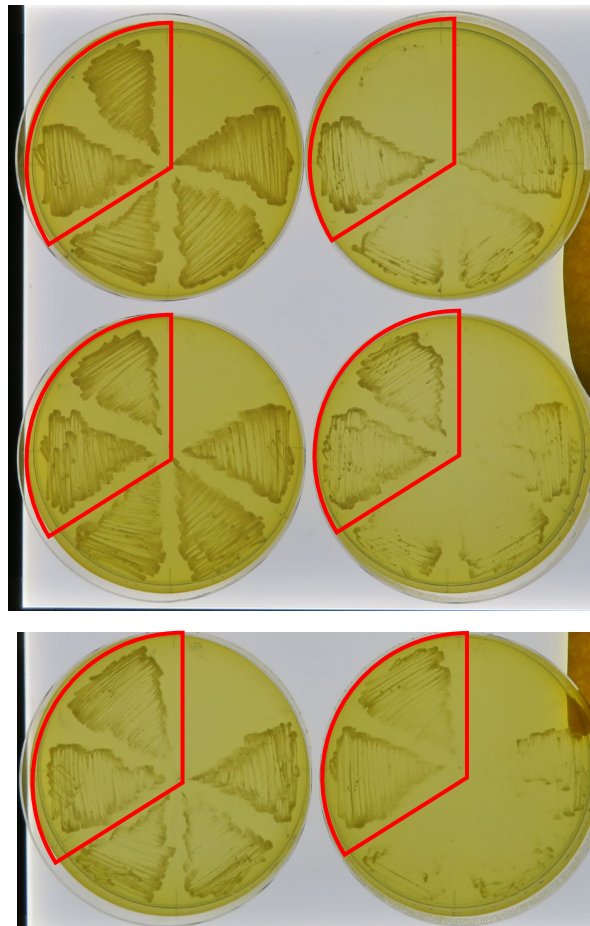

**Fig. 4B**

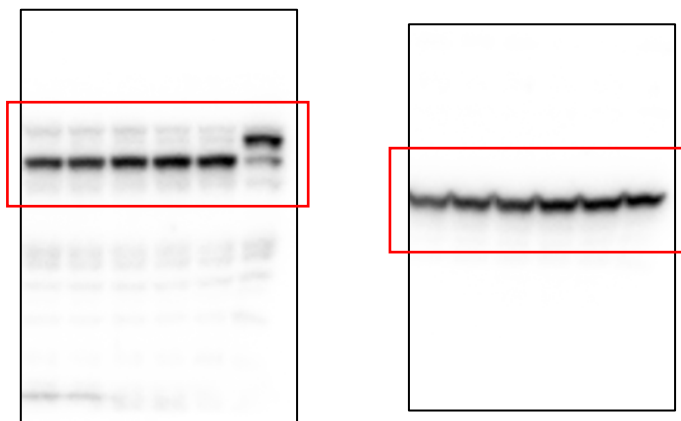

Fig. 4C

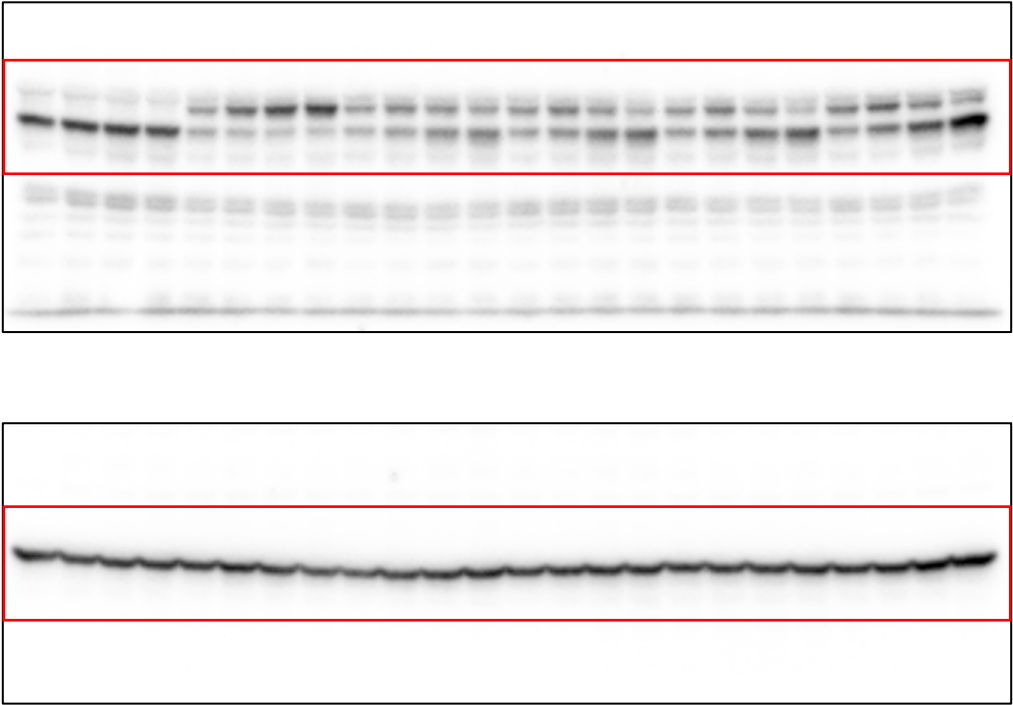

Supplement: Supplementary file 28 [file LSA-2025-03225_SdataF4.1.pdf]

**Fig. S14A**

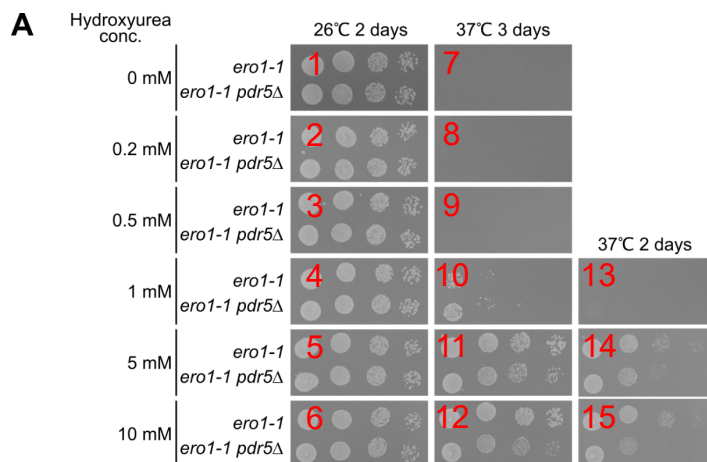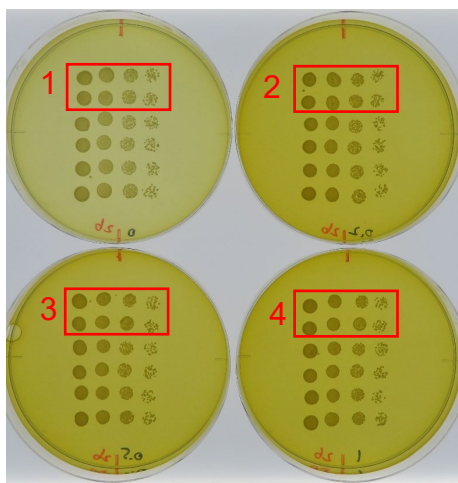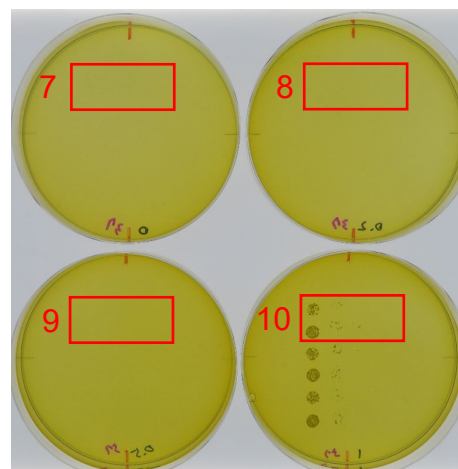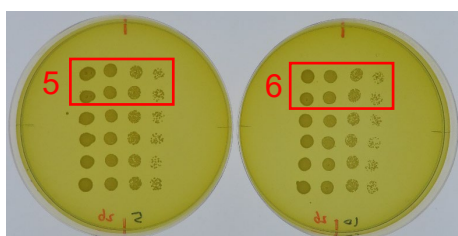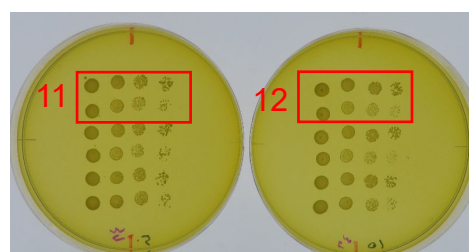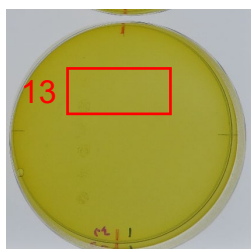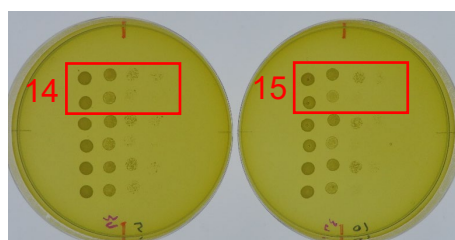

Supplement: Supplementary file 30 [file LSA-2025-03225_SdataFS14.1.pdf]

**Fig. S15**

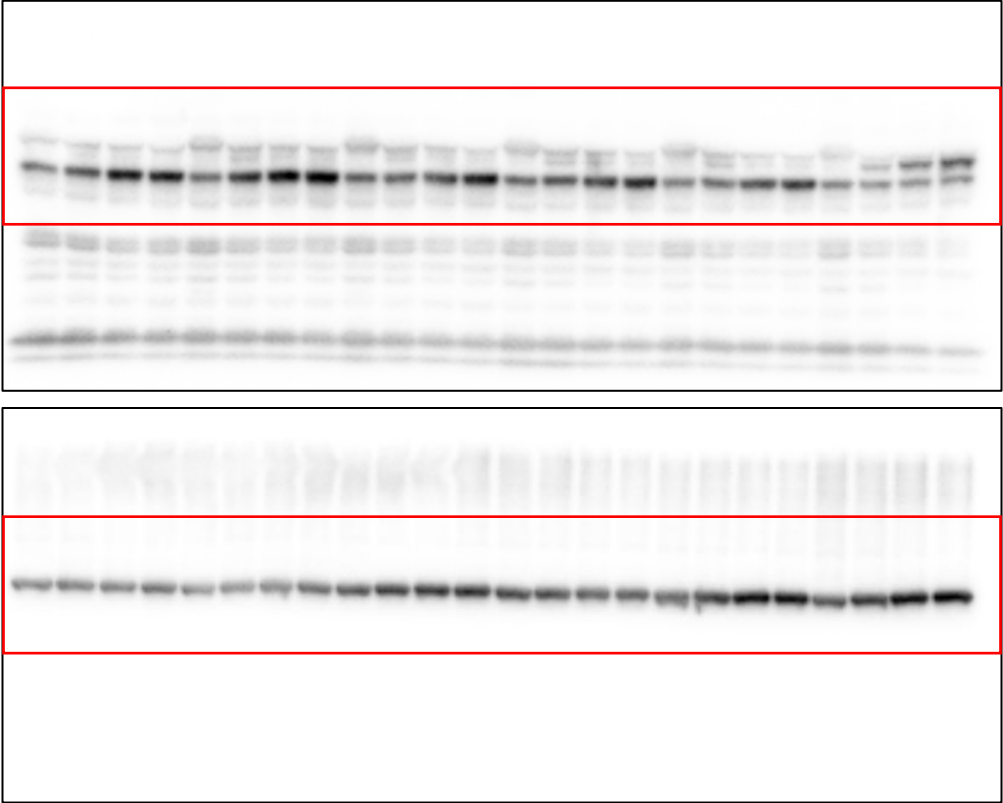

Supplement: Supplementary file 32 [file LSA-2025-03225_SdataFS15.pdf]
