# Supplementary material for: Hydroxyurea modulates thiol–disulfide homeostasis in the yeast endoplasmic reticulum
Source: Life Sci Alliance. 2025 Jun 20;8(8):e202503225. doi: 10.26508/lsa.202503225 (PMC12181674; doi:10.26508/lsa.202503225)
Supplement: Supplementary file 3 [file LSA-2025-03225_SdataFS1.1.pdf]

Fig. S1A

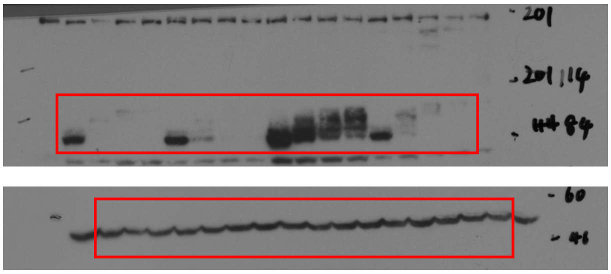

Fig. S1B

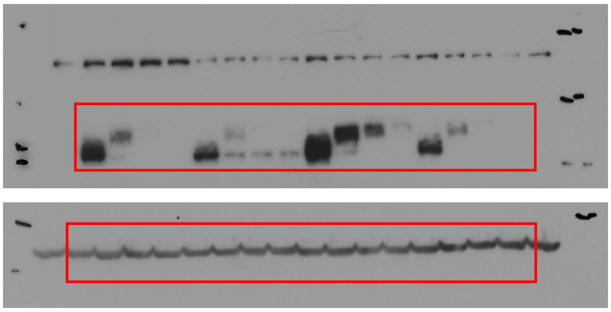

Fig. S1C

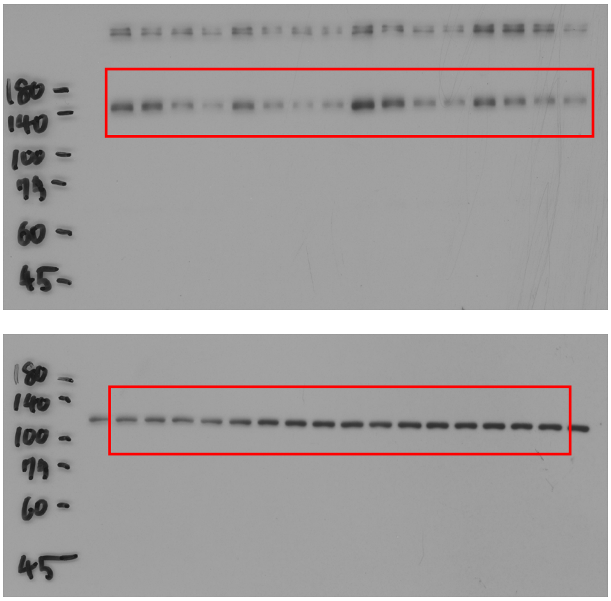

Fig. S1D

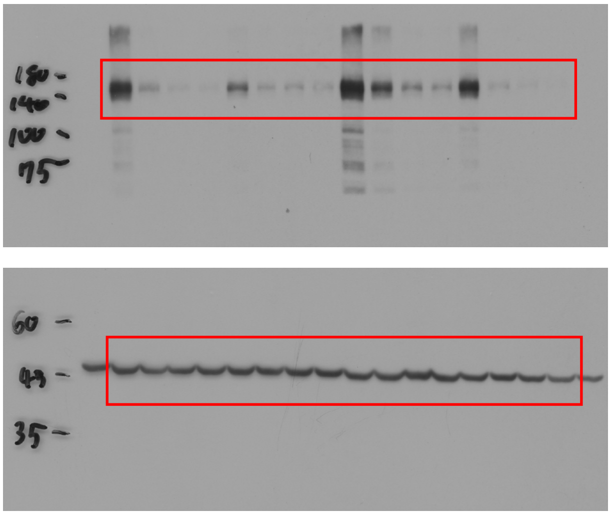

In Fig. S1A and S1B, the membranes were cut prior to hybridization with antibodies.
